# Cell death in skin function, inflammation, and disease

**DOI:** 10.1042/BCJ20210606

**Published:** 2022-08-05

**Authors:** Holly Anderton, Suhaib Alqudah

**Affiliations:** 1 Inflammation division, The Walter and Eliza Hall Institute for Medical Research, Parkville, Melbourne, Victoria, Australia; 2 Department of Medical Biology, University of Melbourne, Parkville, Melbourne, Victoria, Australia; 3 Melbourne Dental School, The University of Melbourne, Carlton, Victoria, Australia; 4 Faculty of Dentistry, Jordan University of Science and Technology, Irbid, Jordan

**Keywords:** apoptosis, cell death, cornification, necroptosis, pyroptosis, skin

## Abstract

Cell death is an essential process that plays a vital role in restoring and maintaining skin homeostasis. It supports recovery from acute injury and infection and regulates barrier function and immunity. Cell death can also provoke inflammatory responses. Loss of cell membrane integrity with lytic forms of cell death can incite inflammation due to the uncontrolled release of cell contents. Excessive or poorly regulated cell death is increasingly recognised as contributing to cutaneous inflammation. Therefore, drugs that inhibit cell death could be used therapeutically to treat certain inflammatory skin diseases. Programmes to develop such inhibitors are already underway. In this review, we outline the mechanisms of skin-associated cell death programmes; apoptosis, necroptosis, pyroptosis, NETosis, and the epidermal terminal differentiation programme, cornification. We discuss the evidence for their role in skin inflammation and disease and discuss therapeutic opportunities for targeting the cell death machinery.

## Introduction

The skin functions as our bodies first line of defence, an interactive barrier, protecting us from a constantly changing environment full of physical, chemical, and biological aggressors. Maintenance of barrier integrity, immune surveillance, and rapid response are fundamental to proper function. The epithelial barrier and immune cells orchestrate this multifaceted protection. Acute and chronic inflammatory skin diseases can arise due to abnormal over-reactions to the changing environment. Given the large variety of commensal microbial species that live on the skin surface and in hair follicles, a high degree of tolerance is required. Maintaining skin homeostasis is immensely complex and involves a highly regulated nexus of interactions between the different cellular compartments and the microbial communities (Box 1 – Structure and key cellular players in the skin).

### Programmed cell death is essential for maintaining and restoring skin homeostasis

Programmed cell death (PCD) plays a vital role in forming and maintaining the cutaneous and mucosal barriers. Maintenance of the protective barrier depends on the balanced process of keratinocyte differentiation and cornification. While the process of cornification is programmed and results in the death of the cells, unlike other forms of PCD, the cornified cells become an integral part of the tissue [1].

PCD also supports the response to and recovery from acute injury and infection. Apoptosis of immune cells is critical for the resolution of acute inflammation associated with injury and infection. Inflammatory forms of cell death such as necroptosis, pyroptosis, and NETosis may have evolved to provide an opposing effect on inflammation. Induction of these cell death programmes is often in response to pathogen-associated molecular patterns (PAMPs) or to self-derived damage-associated molecular patterns (DAMPs), where enhanced inflammatory and immune responses are required to produce an adequate tissue response [2–5]. Less apparent is a potential physiological role for ferroptosis-associated inflammation. This form of cell death appears to be one of the oldest in an evolutionary sense, a way of eliminating cells that are not effectively managing the consequences of our oxygen-based metabolism. The inflammatory consequences of ferroptosis may have been repurposed to provide physiological roles in immune surveillance and tumour suppression [6].

**Box 1. Structure and key cellular players of the skin**

The skin can be broadly broken down into the epidermis, the dermis, and the hypodermis. The hypodermis is the subcutaneous fat layer, it consists of adipocytes in a loose connective tissue and contains larger blood vessels en-route to the dermis. The dermis consists of dermal fibroblasts in an extracellular matrix and forms a tough, irregular connective tissue. Structures such as hair follicles, sweat glands, sebaceous glands, nerves, and sensory receptors are present in this layer, as is blood supply and lymphatic vessels servicing and supporting the skin. Immune cell populations in the dermis, include macrophages, T-cells, mast cells, eosinophils, dendritic cells, and innate lymphoid cells (ILCs).

The outermost layer of the skin is the epidermis, a stratified squamous keratinised epithelium. In the epidermis the main populations of immune cells are CD8^+^ resident memory T-cells (T_RM_) and an epidermal resident population of Antigen Presenting Cells called Langerhans Cells. Keratinocytes are the main structural element of the epidermis; however, they also play a key immunomodulatory role. They are responsible for secreting antimicrobial peptides even in the absence of injury, and stimulating inflammation in response to DAMPs and PAMPs. They are necessarily one of the first participants in the inflammatory process and thus play an important role in antigen presentation and modulation of the immune response. However, as these cells are at the frontline, a degree of tolerance is required. Microorganisms colonise not only the surface of the skin but also into hair follicles, sweat glands and sebaceous glands, meaning that host exposure to microbial species is not limited to the dead cells making up the *stratum corneum*. Epidermal pathologies and activation of inflammatory signalling cascades in the epidermis are common in ISDs, suggesting a breakdown in immune tolerance, and highlighting the importance of keratinocytes in immune regulation in the skin.

### The intertwining outcomes of cell death and inflammation

**Figure 1.**
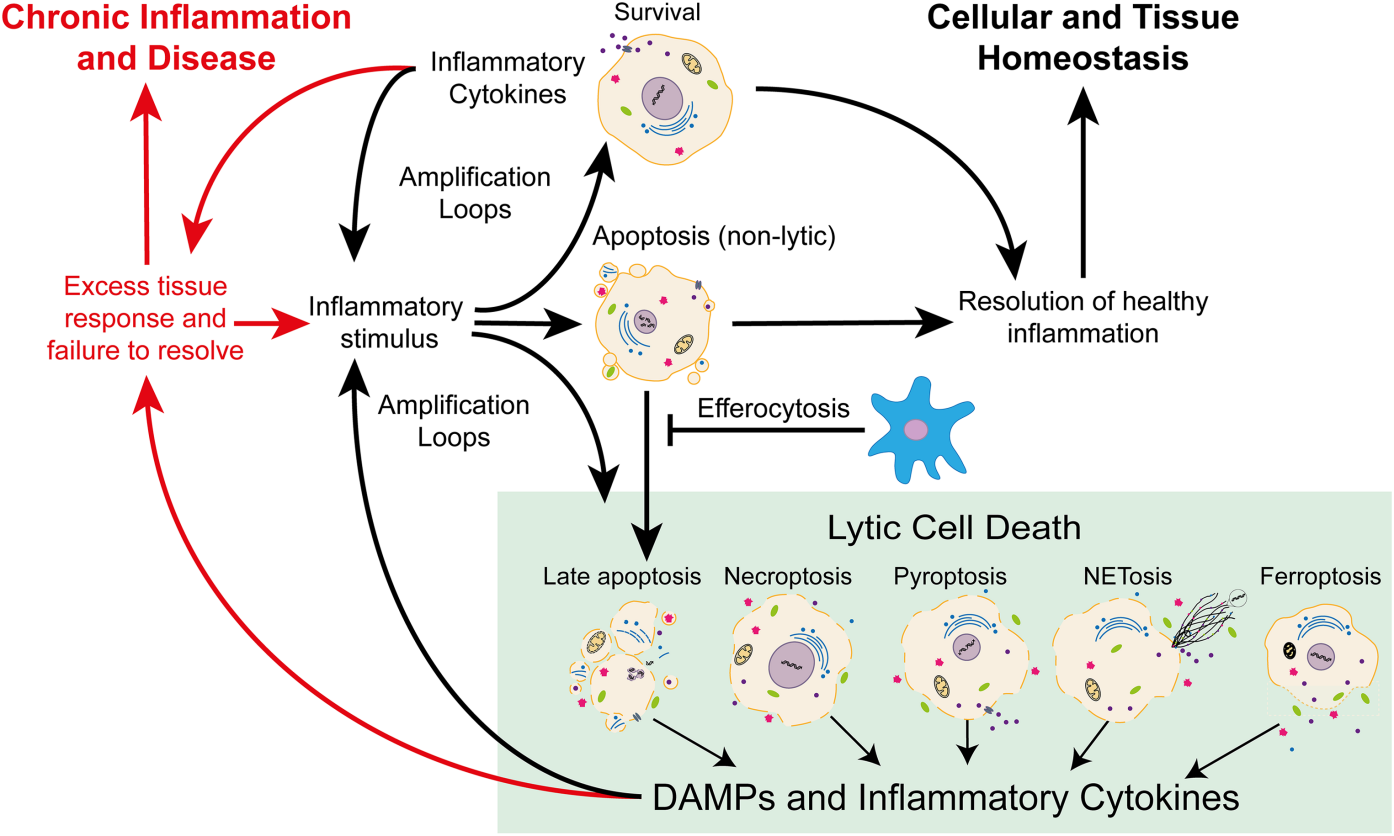
Interactions between inflammatory signals and cell death programmes can enhance both. Acute inflammation is a healthy response to danger signals such as, trauma, stress, or infection. An inflammatory stimuli can prompt several outcomes for a cell. Most often the response will be survival signalling, which enhances the expression of proteins associated with proliferation and inflammation. In some circumstances, an inflammatory stimulus may initiate a cell death pathway. Apoptosis is typically non-inflammatory, and an important part of the resolution of acute inflammation in a tissue, however, apoptotic cells will proceed to late apoptosis when phagocytic clearance (efferocytosis) is delayed. Other lytic forms of programmed cell death, including necroptosis, pyroptosis, NETosis and ferroptosis, are thought to promote inflammation by releasing damage-associated molecular patterns (DAMPs), and other inflammatory mediators, into the extracellular environment. Inflammatory amplification loops play a physiological role in acute tissue inflammation in order to generate adequate tissue and immune responses. Lytic forms of cell death, including late apoptosis, can further enhance inflammatory amplification loops. Once an insult is dealt with, the resolution of inflammation is vital to restore cellular and tissue homeostasis. Excess inflammatory signalling can lead to ongoing amplification cycles that fail to resolve, resulting in chronic inflammation, enhanced cell death, tissue pathology and disease.

Activation of inflammatory signalling pathways is one of the earliest innate immune responses during injury and infection. Extracellular signals detected by cells in the skin initiate acute inflammation. Ligation of signalling molecules to membrane-bound receptors generates internal signalling cascades that can affect cell function by regulating genes and transcription factors. In some circumstances, these signalling molecules can also induce cell death programmes. These differing outcomes are entwined as cell death can also drive inflammatory signalling and immune cell recruitment. While the downstream consequences of inflammation can include the production of death receptor ligands leading to enhanced cell death. Lytic forms of cell death can promote inflammation due to the uncontrolled release of inflammatory cell contents [5,7–9], including pro-inflammatory DAMPs such as HMGB1 [10–16], IL33 [13,17,18], uric acid [19], S100 proteins [20], heat shock proteins (HSPs) [21], and nucleic acids/DNA [22–25]. The interplay between inflammation and cell death can contribute to inflammatory amplification loops required to generate adequate tissue and immune responses during injury or infection. However, when poorly regulated, these interactions can result in a vicious cycle leading to enhanced and ongoing inflammation and disease (Figure 1).

When both inflammation and cell death feature prominently in the pathology of a disease or phenotype, we must consider their relative contributions to pathogenesis. Is cell death driving inflammation, or is inflammation causing excessive cell death? Can we reduce the impact of both by targeting one or the other, or is a multi-pronged approach required? Here we discuss different types of PCD, the inflammatory consequences, and their association with inflammatory skin phenotypes in mice and humans.

## Cell death modalities in skin function and dysfunction

PCD has many distinct forms, several of which are known to contribute to skin inflammation and disease. These include apoptosis, necroptosis, pyroptosis, NETosis, and ferroptosis. Additionally, while the nomenclature committee on cell death suggests that terminal differentiation should be considered distinct from the concept of PCD [26], cornification, which does result in the death of the cell, is integral to proper skin function; and management of alternative cell death programmes is essential to its proper execution. We will be discussing the role of each of these mechanisms in skin homeostasis and inflammation.

### Cornification

**Figure 2.**
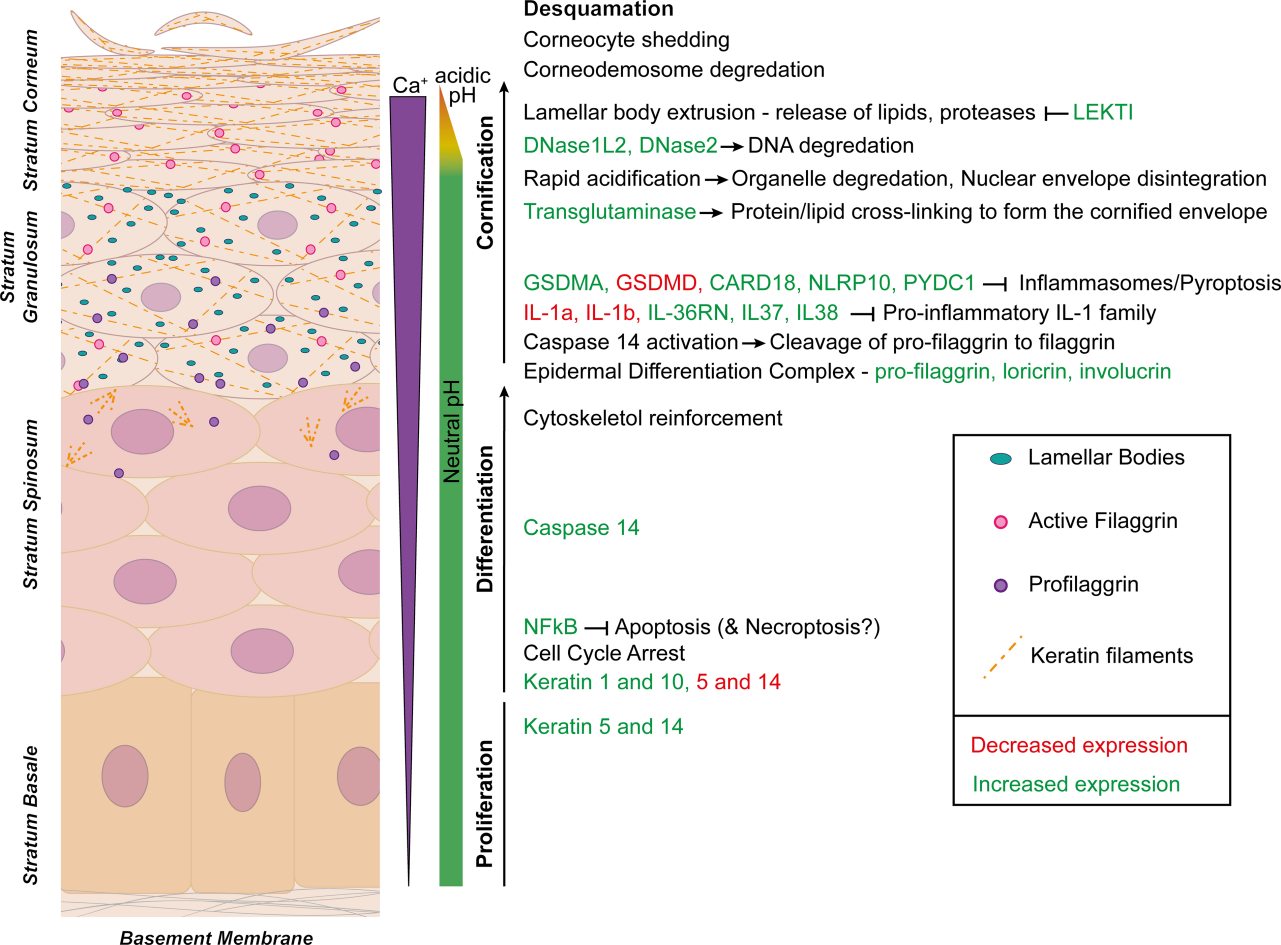
Overview of epidermal differentiation and cornification. The epidermis consists of layers of keratinocytes at different stages of differentiation. Basal keratinocytes, distinguished by the expression of keratins 5 and 14, proliferate, producing new cells that will differentiate while passaging towards the skin surface. Cells of the spinous layer enter cell cycle arrest. They express typical markers of differentiation such as keratins 1 and 10, and caspase-14. NF-κB expression is also increased, inhibiting apoptosis. Pro-filaggrin and proteins of the epidermal differentiation complex are expressed as cells transition from the spinous to the granular layer. Cornification is initiated from the granular layer. Caspase-14 becomes active and contributes to the processing of filaggrin, and various anti-inflammatory proteins are expressed. Lamellar bodies are extruded, releasing serine proteases and lipids that will fill the inter-corneocyte space. Rapid intracellular acidification enables degradation of the nucleus and other organelles, at which point the cells would be considered dead. Keratins and other proteins are cross-linked by transglutaminases forming the cornified envelope. Eventually, proteases degrade the corneodemosomes that hold the corneocytes together allowing the cells to shed during desquamation.

One of the critical functions of the skin is as a mechanical barrier. Cornified keratinocytes in the epidermis primarily perform this function. The epidermis comprises several layers of keratinocytes — at the bottom is the *stratum basale*, a layer of proliferating keratinocytes attached firmly to a contiguous basement membrane separating the dermis from the epidermis [27]. It is from these basal keratinocytes that the cells of the upper layers are derived. Basal keratinocytes passage up through the layers of the epidermis in an ordered path, influenced by calcium gradients, while undergoing terminal differentiation. The first stage of differentiation forms the *stratum spinosum*, typically the thickest layer of the epidermis and the primary location of Langerhans cells (LCs). Further up is the *stratum granulosum*, which consists of flattened, nucleated, and highly granulated cells between which tight junctions form [1,27,28]. The cornification process occurs from this stage. A rapid decrease in intracellular pH facilitates denucleation [29] and complete DNA degradation by the keratinocyte-specific enzyme DNase1L2, leading to lysis of the nucleus and the formation of corneocytes [30]. Corneocytes are flattened, highly keratinised, non-living cells organised in layers to form the external protective layer, the *stratum corneum.* Corneocytes’ intracellular organelles and cell contents are replaced by a proteinaceous layer, ‘the cornified envelope,’ with cross-linking of proteins at the cell periphery [1,27,28,31,32]. As part of normal barrier function, the dead cells of the *stratum corneum* are continuously shed from the epidermal surface [33] (Figure 2). The rate of keratinocyte proliferation in the *stratum basale* must balance the rate of sloughing of the cornified cells of the *stratum corneum* to maintain epidermal homeostasis.

#### Death by cornification does not end cell function

While no further gene expression is possible once the nucleus is degraded, functional proteins are retained and can be activated after cell death. Corneocytes are, therefore, not completely inert and can respond to environmental changes. Their responses, however, are pre-determined based on the suite of functional proteins, including keratins, ceremide, loricrin, and filaggrin preserved during cornification [33,34]. These proteins are responsible for giving strength and elasticity to the epidermis and ensuring imperviousness and water retention.

#### Potential danger signals inherent in cornification are actively suppressed

Cell death during cornification involves processes that have the potential to activate danger signals. Failure to effectively suppress inflammation can invoke further inflammatory signalling and risk disturbing the differentiation process of adjacent KCs. The tightly controlled gene expression programme for cornification, therefore, includes genes encoding proteins that mediate the degradation of potential DAMPs and regulate enzymatic activity [32]. For example, DNase1L2 and DNase2 completely degrade DNA upon nuclear envelope dissolution [30,35]. This is notably different from apoptosis, where DNA is degraded (fragmented) by DNases, but the fragments are retained within the apoptotic cell [36,37]. One reason for this difference may be the differing cell fates. Cornified cells are retained as part of the epidermis for days or weeks, while apoptotic cells are destined for elimination by phagocytes typically within hours of death, at which point they enter lysosomes and the cell remnants, including the fragmented DNA, are fully degraded [37,38].

Cornification requires a massive activation of epidermal proteases. Lamellar bodies released from the granular layer during cornification contain serine proteases that degrade corneodesmosomes and promote desquamation [31,32]. The *SPINK5* gene encodes the serine protease inhibitor LEKTI which regulates the activity of these desquamation-involved proteases [39]. Truncating mutations in *SPINK5* cause Netherton's Syndrome, a rare, autosomal recessive disorder characterised by congenital ichthyosis with defective cornification, bamboo hair, and severe atopic manifestation [39,40].

An interesting gene expression screen also identified proteins explicitly associated with keratinocyte terminal differentiation that do not appear to contribute to the cornification process at all but instead play a suppressive or anti-inflammatory role. These include modulators of pyroptosis (CARD18, NLRP10, PYDC1) and IL-1 family antagonists (IL-36RN, IL-37, IL-38) [32,41].

## Apoptosis

Apoptosis is a highly regulated process that plays a vital role in restoring and maintaining skin homeostasis by eliminating unnecessary or damaged cells. It also plays a key role in hair follicle renewal and is critical to ensuring immunologic tolerance. There are two distinct apoptotic pathways, the intrinsic or mitochondrial pathway and the extrinsic or death receptor pathway. Although the initiating steps in intrinsic and extrinsic apoptosis differ, the pathways converge upon the activation of a family of proteases called caspases [26]. The process downstream of caspases is highly regulated and results in the packaging of cellular components into apoptotic bodies prior to cell degradation without the release of cell contents [42]. Thus, apoptosis is dogmatically considered to be non-inflammatory and immunologically silent.

### Intrinsic apoptosis

**Figure 3.**
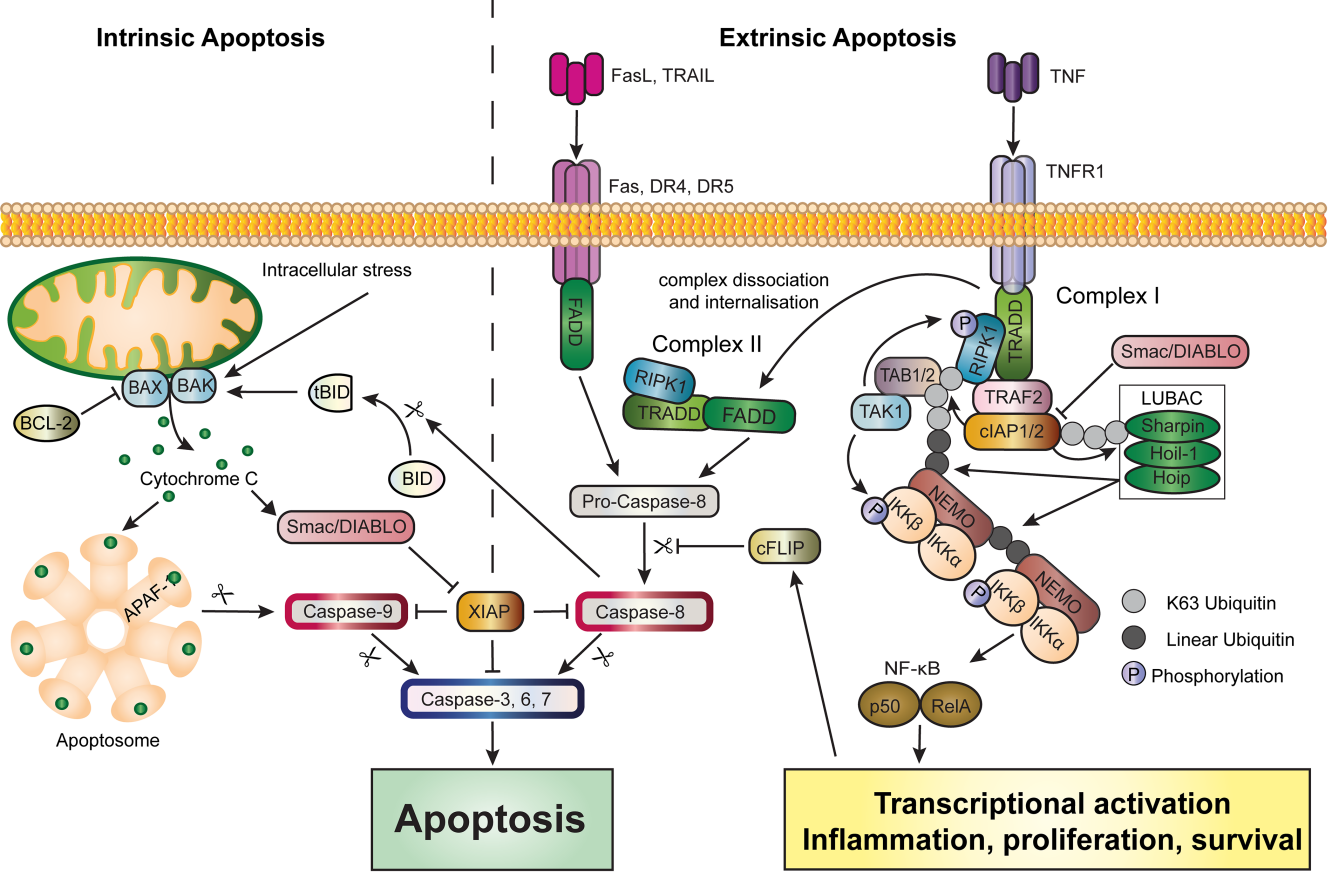
Overview of intrinsic and extrinsic apoptosis. (**A**) Intrinsic Apoptosis — intracellular stress activates pro-apoptotic members of the BCL2 family, BAX and BAK, which form pores in the mitochondrial outer membrane. Cytochrome-c is released into the cytosol where it binds to the adaptor protein APAF1 to promote the formation of the Apoptosome. This molecular platform facilitates caspase-9 activation, initiating the caspase cascade. Executioner caspases can then cleave hundreds of substrates limiting immunogenicity during apoptosis. Cytochrome-c is not the only protein released from the mitochondria upon membrane permeabilization. The IAP antagonist protein Smac/DIABLO is contained in the mitochondria and is released during intrinsic apoptosis. Smac/DIABLO antagonises XIAP, lifting the late-stage inhibition of caspases, and ensuring execution of the caspase cascade. (**B**) Extrinsic apoptosis can occur following death receptor ligation (Fas, TRAIL or TNF) and activation of the apoptotic caspase cascade, beginning with caspase-8. However, ligation of TNFR1 will preferentially activate pro-survival signals. TNF binding promotes the formation of complex I via recruitment of TRADD to the receptor's death domain. TRAF2, RIPK1 and the cIAPs can then be recruited to the receptor complex. The cIAPs recruit LUBAC, and mediate ubiquitination of RIPK1. This leads to the recruitment of a TAK1-containing complex, enabling subsequent recruitment of NEMO and the IKKs. Phosphorylation of RIPK1 via the IKKs and TAK1, prevents the formation of RIPK-dependent death complexes. Activation of canonical NF-κB can then proceed, promoting transcription of pro-inflammatory genes and genes encoding anti-apoptotic factors such as cFLIP. Ongoing activation of TNFR1 or defects in complex I regulatory components will promote dissociation and internalisation of the receptor complex to form a cytosolic death complex, known as complex II. The interaction of complex II components, enables oligomerisation and autoproteolytic cleavage of Caspase-8, initiating caspase-mediated apoptosis. Activated caspase-8 can also cleave BID to its truncated form (tBid) which can activate BAK initiating, intrinsic apoptosis. The subsequent release of Smac/DIABLO can lift the XIAP break on caspase activation ensuring completion of the death programme. Smac/DIABLO also antagonises cIAP1 and 2, thus, while intrinsic and extrinsic apoptosis operate independent of each other, they also each include mechanisms to promote activation of the other.

Intrinsic apoptosis occurs in response to disruptions of cellular homeostasis, such as oxidative stress or DNA damage [26]. However, it is also induced in healthy cells as part of their natural life cycle. Intrinsic apoptosis is characterised by mitochondrial outer membrane permeabilisation (MOMP). Upon activation, pro-apoptotic members of the BCL2 family, such as BAX and BAK, oligomerise into pore-forming complexes on the mitochondrial outer membrane [43,44]. These pores enable the release of pro-apoptotic proteins, including cytochrome-c and the inhibitor of apoptosis protein (IAP) inhibitor Smac/DIABLO [45–47], into the cytosol. Cytosolic cytochrome-c associates with the adaptor protein APAF1 to form the apoptosome, a molecular platform that facilitates the activation of caspase-9, triggering the caspase cascade [26] (Figure 3A). While disruption of the mitochondrial membrane is enough to kill a cell, the apoptotic caspase cascade, which occurs well after the point of no return, functions to prevent type I interferon production [42,48] and ensures that DNA and other intracellular substrates with the potential to act as DAMPs are cleaved and inactivated [38,42,48,49].

Defects in the regulation of intrinsic apoptosis can affect the hair follicle cycle, causing poor hair growth, premature greying, and alopecia [50]. Anti-apoptotic Bcl-2 is highly expressed in melanocyte stem cells, specifically protecting them from apoptosis during catagen. Bcl-2 deficiency in mice causes their apoptotic elimination resulting in premature hair greying [50,51]. Conditional knockout of Bcl-2 in mouse epidermis or systemic treatment with the Bcl-2 antagonist ABT-199/venetoclax during catagen causes selective loss of hair follicle-associated stem cells leading to disrupted hair follicle regeneration and delayed hair regrowth [52].

### Extrinsic apoptosis

Extrinsic apoptosis is initiated upon detection of an extracellular signal, either cytokines produced by other cells locally or systemically or DAMPs or PAMPs that activate pattern recognition receptors (PRRs) [26,53]. Initiation is most often by ligation of death receptors, such as TNFR1, FAS, or TRAIL receptors 1 (TRAILR1/Death Receptor (DR)4) and 2 (TRAILR2/DR5), with their corresponding ligands (FASL, TRAIL, and TNF, respectively) [26] (Figure 3B). However, it can also be triggered upon activation of Toll-Like Receptors (TLRs) 3 and 4 via TIR domain-containing adapter-inducing interferon-β (TRIF) mediated receptor complex formation, usually during infection [53] (Figure 3B).

Death receptor–ligand binding initiates the formation and internalisation of death-inducing signalling complexes (DISCs). In Fas and TRAIL-mediated apoptosis, receptor activation enables binding of Fas-associated death domain (FADD) to the receptors’ own death domains. The death effector domain (DED) of FADD can then bind pro-caspase-8 triggering its autoproteolytic cleavage and oligomerisation to form active caspase-8, initiating the caspase cascade [54,55].

Activation of TNFR1 initiates recruitment of TNFR1-associated death domain protein (TRADD), RIPK1, and TRAF2 [56–58] to a membrane-bound signalling platform referred to as complex I. TRAF2, in turn, recruits the E3 ubiquitin ligases, cellular inhibitor of apoptosis proteins (cIAP)1 and cIAP2 [59]. The cIAPs conjugate K11, K48 and K63 ubiquitin to RIPK1, which sequesters RIPK1 at the receptor complex [60–62]. The K63 ubiquitin on RIPK1 acts as a scaffold for recruitment of the transforming growth factor-β-activated kinase 1 (TAK1)/TAK1-binding protein (TAB)1/2 complex and the IκB kinase (IKK) complex [58,63,64]. The cIAPs also recruit the linear ubiquitin-chain assembly complex (LUBAC) to complex I. LUBAC consists of three proteins, SHANK-associated RH domain-interacting protein (SHARPIN), hemeoxidized iron-regulatory protein 2 ubiquitin ligase-1 (HOIL-1), and HOIL-1-interacting protein (HOIP). LUBAC assembles linear ubiquitin chains on complex I components including RIPK1 and the IKK regulatory subunit, nuclear factor-kappa B (NF-κB) essential modulator (NEMO). This further recruits the NF-κB catalytic subunits IKK1/IKKα and IKK2/IKKβ [65–68] (Figure 3B).

Activated IKK mediates phosphorylation and degradation of Inhibitor of NF-κB (IκB) proteins resulting in the activation of NF-κB and expression of NF-κB dependent genes. These include genes encoding anti-apoptotic proteins such as the caspase-8 regulator, cellular FLICE-like inhibitory protein (cFLIP) [58,63]. cFLIP is structurally related to caspase-8 but lacks proteolytic activity. The presence of cFLIP constrains caspase-8 activation, limiting substrate cleavage to only a subset of proteins located within the complex, including RIPK1 (Figure 3B) [58,63]. An alternative cFLIP isoform (cFLIP_long_) can prevent caspase-8 mediated substrate cleavage entirely, thereby blocking apoptosis but also, by failing to cleave RIPK1, promoting the formation of the necrosome (discussed later) [69].

The post-translational regulation of RIPK1 plays an essential role in shifting the response from cytokine production to cell death upon activation of TNFR1 [70–72]. Within complex I, TAK1 and the IKKs will phosphorylate RIPK1 preventing activation [73] and limiting downstream death signalling [74]. Reduced ubiquitylation of RIPK1 favours dissociation and internalisation of complex I, and recruitment of FADD, pro-caspase-8, and RIPK3, forming a RIPK1-dependent complex known as complex II (Figure 3B) [73].

The interaction of components in complex II, including activated RIPK1, prompts oligomerisation and autoproteolytic cleavage of pro-caspase-8 to its active form. Disruption of complex I components, such as TAK1, TRAF2, cIAPs, or LUBAC, affects post-translational regulation of RIPK1 [70–72] and reduces NF-κB activation resulting in low expression of cFLIP. Reduction in cFLIP levels leads to complete activation of caspase-8 [58,63] and initiation of the caspase cascade.

### Caspase cascades prevent excess inflammation during intrinsic and extrinsic apoptosis

Both intrinsic and extrinsic apoptosis conclude with the initiation of a caspase cascade. Initiator caspases (e.g. caspase-8 and -9) cleave and activate executioner caspases (e.g. caspase-3, -6, and -7). X-linked inhibitor of apoptosis protein (XIAP) is a direct inhibitor of caspases-3, -7, and -9 and acts as a final break before the execution of apoptosis [75–77]. It is inhibited by the mitochondrial protein (Smac/DIABLO), which is released from the mitochondria upon the release of cytochrome-C during MOMP [46,78,79].

Once activated, the executioner caspases cleave hundreds of substrates, resulting in enzymatic degradation of organelles, DNA fragmentation, and membrane blebbing [42]. Activation of caspases instigates mechanisms that actively suppress potentially inflammatory responses. For example, the apoptotic caspase cascade functions to prevent type I interferon production during Bax/Bak dependent apoptosis [48], and caspases-3 and -7 have been shown to inactivate IL-33 [80]. Genomic DNA is cleaved into small fragments [37], and the prototypical DAMP, HMGB1, remains bound to chromatin [14] limiting its inflammatory potential until a late stage of apoptosis following DNA fragmentation [81]. Exposure of phosphatidylserines on the cell surface act as ‘find me’ and ‘eat me’ signals to attract phagocytes and prompt the rapid consumption of the dying cell in a process known as efferocytosis [82]. This, in theory, should limit activation of PRR pathways in surrounding cells, as it prevents the release of cell contents capable of provoking inflammation.

### Apoptosis in normal skin function

Apoptosis plays an essential role in maintaining and restoring normal skin function. For example, hair follicles are a complex mini-organ of the skin that host the stem cell microenvironment. Maintenance of hair follicles is fundamental for skin homeostasis and hair growth and for efficient initiation of tissue repair [83,84]. Hair follicle renewal involves cycling through a growth phase (anagen), apoptosis-driven hair growth recession (catagen), a resting phase (telogen), and shedding (exogen) [84]. Defects in the regulation of intrinsic apoptosis can affect the hair follicle cycle, causing poor hair growth, premature greying, and alopecia [50]. Anti-apoptotic Bcl-2 is highly expressed in melanocyte stem cells, specifically protecting them from apoptosis during catagen. Bcl-2 deficiency in mice causes their apoptotic elimination resulting in premature hair greying [50,51]. Conditional knockout of Bcl-2 in mouse epidermis or systemic treatment with the Bcl-2 antagonist ABT-199/venetoclax during catagen causes selective loss of hair follicle-associated stem cells leading to disrupted hair follicle regeneration and delayed hair regrowth [52].

Defective apoptosis also leads to an impairment of negative selection in the thymus and the consequent persistence of autoreactive T-cells and B-cells that drive inflammation throughout the body, contributing to autoimmune disease. FAS polymorphisms that lead to defective apoptosis contribute to systemic lupus erythema (SLE) pathogenesis, a systemic disease that commonly presents with chronic inflammatory cutaneous manifestations [85].

In sunburn, UVB-mediated activation of p53 up-regulates pro-apoptotic proteins in basal keratinocytes leading to cell cycle arrest and epidermal apoptosis. This is critical for eliminating DNA-damaged cells to prevent skin carcinogenesis [86–88].

Apoptosis is also essential in the cutaneous wound repair process. Apoptosis of neutrophils and inflammatory cells is critical for the resolution of acute inflammation and can start as early as 12 h post-injury [89–91]. During tissue recovery, immune cell apoptosis and efferocytosis below the healing wound edge signal inflammation down-regulation and trigger proliferative responses in keratinocytes, prompting wound closure and re-epithelialisation [92,93]. Then during remodelling, myofibroblasts apoptosis is initiated to eliminate granulation tissue and restore pre-wound structure [90,91]. Defects in apoptosis can prolong inflammatory responses and delay tissue regeneration and remodelling, leading to poorly-healing wounds, fibrosis, and excessive scarring [90,92,94].

### When apoptosis is not so silent

Millions of cells die by apoptosis in the average human every day. Apoptotic cell death must, therefore, operate in a non-inflammatory manner. The caspase cascade functions to minimise inflammation during apoptosis by preventing activation of inflammatory responses within the cell and reducing the immunogenic potential of cell contents [14,38,48]. However, the assumption that all apoptosis is inherently immunologically silent is an oversimplification. Rapid clearance of apoptotic cells is important for limiting immunogenicity. Efferocytosis typically occurs very early after the initiation of cell death, before membrane permeabilisation, thereby limiting the leakage of intracellular contents. Delayed engulfment allows cells to progress to late apoptosis. Late apoptotic cells will begin to lose membrane integrity resulting in the release of DAMPs and further inflammatory signalling [14,16,95–97].

Apoptosis in tissues can become inflammatory when excessive, as it pushes the limit of local phagocytes to clear the dying cells effectively. Indeed, this may be the function of late apoptotic loss of membrane integrity. Recent work identified NINJ1 as an important mediator of plasma membrane rupture in some forms of PCD. In its absence, BMDMs treated with the chemotherapeutic agents venetoclax or cisplatin to induce apoptosis had reduced indicators of membrane rupture despite cell non-viability, suggesting that the late apoptotic membrane permeabilisation is not simply the passive necrotic breakdown of a dead cell, but can be an active process [98]. The release of cell contents during late apoptosis can indicate at a tissue level the insufficiency of local phagocytes and send the message that systemic reinforcements are needed to clear apoptotic cells, while the previous processing of cellular components by caspases to limit immunogenicity avoids an excessive response. Unfortunately, in some circumstances, that inflammation may also drive further cell death, exacerbating the problem when efferocytosis is already insufficient. In such cases, the inflammatory situation may not be fully resolved without addressing the underlying problem by limiting excessive apoptosis.

Disposal of apoptotic cells in barrier tissues such as the skin can also pose unique problems. Rapid removal of dying cells, without a mechanism to seal the gap, may be more damaging to barrier integrity than retaining a dying cell in place for as long as possible. If the epidermal barrier is breached, commensal and pathogenic bacteria can invade, unleashing an intense inflammatory response. By analogy, a crumbling brick in a wall may function better than no brick at all. Retention of apoptotic keratinocytes can be seen in sunburn, where apoptosis leads to the formation of sun-burn cells (SBCs). These are clearly dead, with pyknotic nuclei and eosinophilic cytoplasm, but are retained in place following the execution of apoptosis, detectable up to 36 h after UVB exposure [99]. While retention of such cells may be useful in the short term, ongoing delays in engulfment will eventually result in late apoptosis and enhanced inflammation.

### Excess epidermal apoptosis drives inflammation in mouse models

Despite its generally being an inflammation moderating force, several mouse models highlight the inflammatory potential of poorly regulated or excessive apoptosis in the skin. Differentiating epidermal KCs appear primed to die, and the active suppression of apoptosis is necessary to maintain barrier integrity. Complete loss of apoptosis inhibition is devastating, resulting in widespread loss of the epidermal barrier. Tamoxifen-induced epidermal loss of cFLIP or pharmacological depletion of the IAPs, cIAP1, cIAP2, and XIAP, result in a complete loss of apoptosis inhibition, causing widespread keratinocyte death and severe, acute dermatological disease resembling toxic epidermal necrolysis (TEN) [100,101]. In both the cFlip and IAP depletion models, the disease pathology is excessive apoptotic keratinocyte death, leading to epidermal necrosis.

The body of evidence supports the idea that reduced expression of NF-κB-dependent genes and thus reduced inhibition of apoptosis allow spontaneous KC apoptosis to occur. Genetic disruption of receptor signalling to NF-κB induces inflammatory skin phenotypes in mice. While there is a diversity of manifestations, including the age of onset and localisation, all are to some degree characterised by epidermal hyperplasia, keratinocyte cell death, infiltration by immune cells, and production of pro-inflammatory cytokines. Rapid elimination of apoptotic cells risks compromising barrier integrity, increasing exposure of metabolically active cells to PAMPs, resulting in increased inflammation. Retention, on the other hand, may support the maintenance of the barrier, but as cells progress to late apoptosis, cell lysis and release of DAMPs can also drive tissue inflammation.

There are several genetic models that demonstrate the inflammatory potential of epidermal apoptosis. Epidermal loss of cIAP1 combined with ubiquitous cIAP2 produced mice with severe neonatal skin inflammation and widespread epidermal apoptosis that was lethal by day 10 postpartum. The loss of one allele of Ripk1 limited lesion formation and significantly extended the lifespan of the mice [100].

*Traf2* EKO mice develop epidermal hyperplasia and skin inflammation from around 10 weeks of age, which can be delayed by genetic deletion of *Tnf* [102]*.* The combined loss of mixed lineage kinase domain-like (MLKL) and caspase-8, but not MLKL alone, prevented TNF-dependent cutaneous disease, demonstrating that apoptotic cell death is a driver of the inflammatory phenotype.

Another genetic model demonstrating the inflammatory potential of epidermal apoptosis is the chronic proliferative dermatitis mutation (*cpdm*) in the LUBAC component, *Sharpin*. This spontaneous loss-of-function mutation results in a progressive dermatitis phenotype that shares clinical and histopathological features with chronic eczema and psoriasis [103,104]. The mutation results in reduced activation of NF-κB and AP1-dependent genes from TNF, CD40L, and TLR signalling pathways and sensitises cells to TNF-induced death [65,105,106]. Extensive genetic experiments have shown that SHARPIN deficient keratinocytes are sensitised to TNFR1 induced, caspase-8 mediated apoptosis and that it is this process that drives the dermatitis [104,107]. Recent work identified LCs as a potential cellular source of pathogenic TNF driving apoptotic cell death in the *Sharpin* mutant mice [108].

## Necroptosis

In contrast with apoptosis, where regulatory mechanisms have evolved to limit inflammation, necroptosis occurs independent of caspases [26]. It causes permeabilisation of the plasma membrane and early cell lysis without protective mechanisms to limit immunogenicity. The process is highly inflammatory as it releases intracellular contents that function as DAMPs [9,109]. Necroptosis is triggered by many of the same stimuli as apoptosis, including death receptor ligation, DNA damage, and infection. The latter may be of particular importance as the lack of cell content processing, and thus the greater inflammatory potential of necroptosis, can promote a more robust tissue response. Something that may be required to effectively combat infection. The trade-off is an increased propensity towards excessive inflammation, with disruptions in regulation of necroptosis potentially predisposing towards the development of inflammatory disease.

### TNF-mediated necroptosis

**Figure 4.**
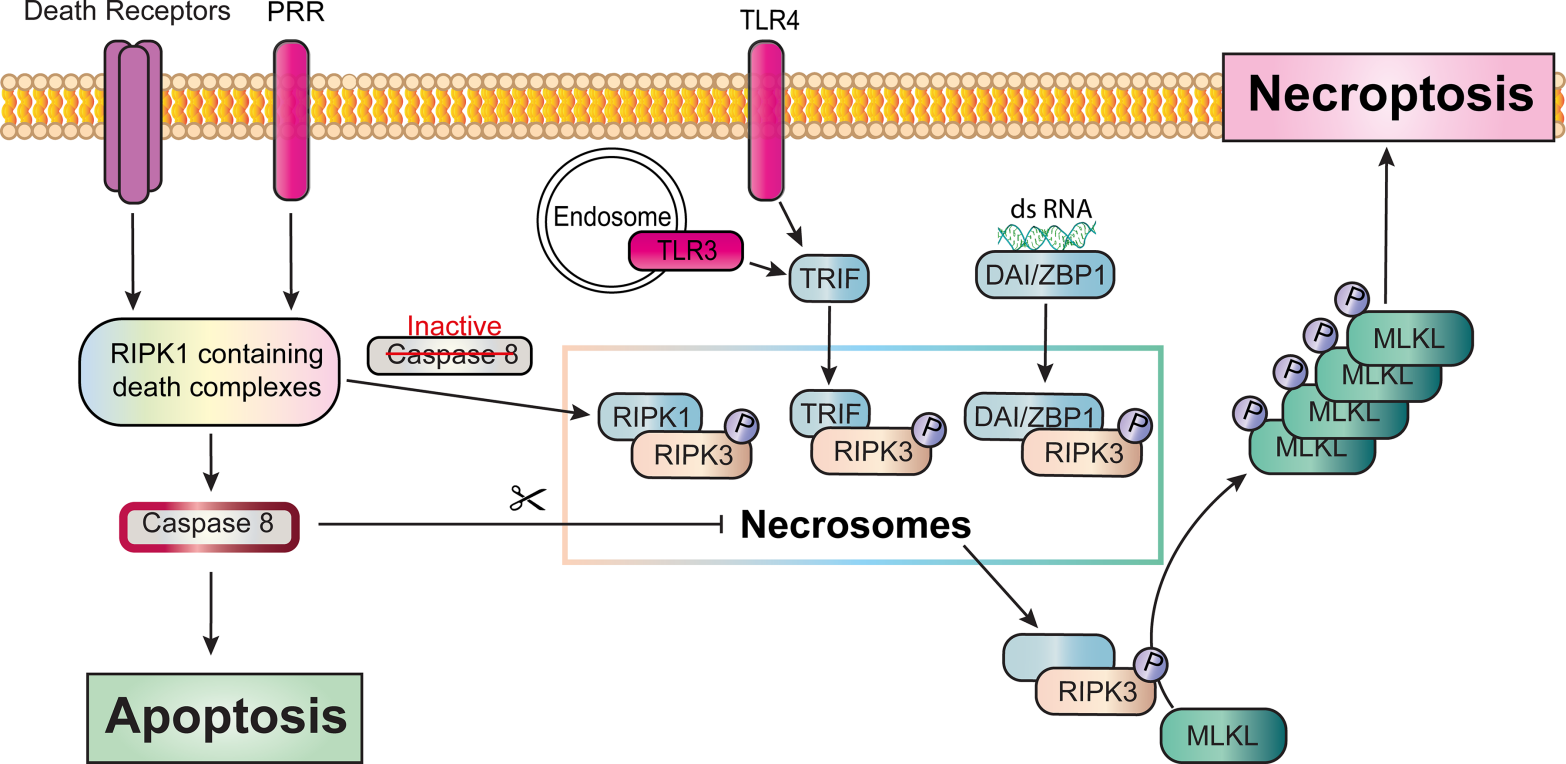
Overview of necroptosis. Downstream of TNFR1, following the formation and dissociation of complex II, activated caspase-8 will cleave RIPK1 and possibly RIPK3, preventing the formation of a second cytolosolic complex, the Necrosome. RIPK1 containing death complexes can also form downstream of FAS and TRAIL and the pattern recognition receptors. Complete inhibition of caspase-8 enables recruitment and phosphorylation of RIPK3 forming the necrosome. Necrosomes can also form independent of RIPK1 via complexes involving other RHIM domain-containing proteins such as ZBP1 upon detecting dsRNA within the cell, or TRIF via endosomal TLR3 or membrane-bound TLR4. Phosphorylated RIPK3 phosphorylates and activates MLKL. Activated MLKL oligomerises and translocates to the cell membrane, generating pores that result in the release of cell contents, cellular swelling and rupture, and the highly inflammatory necroptotic death of the cell.

Complex II formation downstream of TNFR1 activates caspase-8 and causes apoptotic cell death. Activated caspase-8 also cleaves RIPK1 and potentially RIPK3 [110], preventing their oligomerisation and auto-phosphorylation within a second cytosolic complex called the necrosome. Necroptosis is, therefore, not only caspase-independent, missing the protective mechanisms of the caspase cascade, but also caspase inhibited. Complete disruption of caspase-8 activity or increased levels of RIPK3 and the pseudokinase, MLKL, can promote the formation of the necrosome. Within this complex, RIPK3 binds to the RHIM domain of RIPK1 and phosphorylation events stabilise the association and prompt activation of RIPK3 [111–113]. Uncleaved phospho-RIPK3 phosphorylates MLKL, which then oligomerises and translocates to the cell membrane. MLKL-mediated disruption of the plasma membrane results in the release of cell contents, cellular swelling and rupture and necroptotic cell death [9,114,115] (Figure 4).

The precise mechanisms by which oligomerised MLKL causes membrane permeabilisation is a subject of much enquiry but remains somewhat unclear. Two earlier studies suggested that MLKL oligomers could form cation channels facilitating changes in osmotic gradients leading to cellular swelling and eventual membrane rupture [116,117]. However, recent work on Ninj1 showed that osmotic swelling alone is insufficient to prompt membrane rupture at least in the case of the gasdermin D (GSDMD) pore (discussed later in pyroptosis) [98]. Higher order MLKL structures consistent with the formation of a membrane-spanning pore have not been identified. However, it has been reported that oligomerised pMLKL co-accumulates with tight-junction proteins to form plasma membrane ‘hot spots’ during epithelial cell necroptosis [9,118]. Once a threshold density of these spots is reached the membrane ruptures, resulting in necroptotic death. Interestingly, these pMLKL-tight-junction-associated hotspots accelerated necroptosis in neighbouring cells, prior to membrane rupture, suggesting that MLKL-mediated membrane damage can be propagated between cells so long as those cells are also primed to undergo necroptosis [118].

The direct cleavage of RIPK1 by caspase-8 was initially thought to be a mechanism to inhibit necroptosis. However, targeted mutation of RIPK1 to prevent caspase-8 mediated cleavage caused accumulation of RIPK1 and increased stability of the apoptotic death-inducing complex. The result was enhanced apoptosis rather than the formation of the necrosome [119–121]. This supports the idea of a hierarchy of responses in TNFR1 signalling. Signalling to NF-κB activation promotes cell survival and is generally the first response; however, ongoing cellular stress or targeting of complex I signalling components by, for example, infectious agents diverts signalling to initiate cell death [71,122,123]. Apoptosis is the preferred mechanism as it controls the release of cell contents and prevents excessive inflammation. Cleavage of RIPK1 inhibits its kinase activity, preventing necroptosis as hypothesised but does not affect its scaffold function as required for apoptosis. So long as it remains possible, the cell favours apoptosis over necroptosis even when kinase function is restored.

If apoptosis cannot proceed, then necroptosis is activated to ensure cell death occurs. In this case, the death of the cell is more important than the prevention of inflammatory consequences. Indeed, necroptotic cell death, despite being highly inflammatory, can reduce overall tissue inflammation by removing damaged or infected cells that might otherwise continue to produce large amounts of inflammatory mediators [100,124].

### Alternative initiation mechanisms for necroptosis

While TNF-mediated necroptosis is the best understood necroptotic pathway, cytosolic complexes involving RIPK1 also occur downstream of TRAIL and FAS [125–127] as well as PRRs [128,129]. Necroptosis can also be initiated independently of RIPK1 via complexes involving other RHIM domain-containing proteins such as Z-DNA-binding protein 1 (ZBP1, also known as DAI) and TIR domain-containing adapter protein inducing IFNβ (TRIF) [130–132]. These RIPK1-independent necroptotic pathways may be cell-type restricted. They have been observed *in vitro* in endothelial cells and fibroblasts. However, in human immortalised keratinocytes (HaCaT cells) and in macrophages, TLR3-induced necroptosis requires the formation of the RIPK1-RIPK3 necrosome [128,133].

### Necroptosis in mouse models of skin inflammation

As disruption of TNFR1 signalling can favour initiation of not only apoptosis but also necroptosis, there are unsurprisingly several genetic mouse models in which TNFR1-mediated necroptosis has been identified as a driving force behind the inflammatory phenotypes.

Epidermal knockouts (EKO) of the IKK subunits, *Nemo or Ikk2,* develop severe, widespread dermatological phenotypes shortly after birth. *Nemo* EKOs die between postpartum day (P)7 and P10, while *Ikk2* EKOs are lethal by P9 [134–136]*.* Mice with combined epidermal loss of the NF-κB subunits, RelA and c-Rel, also developed inflammatory skin lesions similar to the *Ikk2* EKOs. *Tnfr1* KO rescues these inflammatory phenotypes with mice surviving into adulthood [136,137]. Epidermal-specific KO of TNFR1 was sufficient to rescue the IKK2 EKOs showing again that it is TNFR1 signalling in keratinocytes driving disease [134,138]. Interestingly in the *Ikk2* EKOs, blocking apoptosis by crossing to *Fadd*^EKO^ worsened dermatitis while crossing to *Ripk3^−/−^* or *Mlkl^−/−^* to block necroptosis reduced the severity of the early disease. The necroptotic KOs went on to develop progressive skin lesions from 2 months of age. Blocking both apoptosis and necroptosis by crossing the *Ikk2*^EKO^ to *Fadd^EKO^* and *Ripk3^−/−^* completely prevented the phenotype. These experiments elegantly demonstrated that keratinocyte death by both apoptosis and necroptosis is responsible for the skin inflammation in these mice [134]. Similarly, in the *Rela^EKO^cRel^EKO^* crossing to *Mlkl^−/−^* mice prevented the early onset disease with mice developing inflammatory skin lesions in adulthood instead [134]. The greater disease severity when necroptosis is functional reflects the catastrophic inflammatory potential of this lytic form of cell death. The milder but progressive skin disease when necroptosis is blocked but apoptosis remains functional highlights how low-level but constant apoptotic death in the epidermis can drive chronic inflammation and disease.

Epidermal-specific disruption of RIPK1 binding functions by complete ablation or by RHIM domain mutation also promotes necroptosis-mediated skin inflammation via spontaneous ZBP1 sensing of nucleic acids [22,131,132,139]. While *Ripk1^EKO^* mice develop skin lesions from 1 week after birth, crossing of these mice to mice with two RHIM domain-specific mutations in *Zbp1* (*Zbp1^Za1a2/Za1a2^*) or to *Mlkl^EKO^* mice delayed onset of skin pathology to after 12 weeks of age [22]. This demonstrates a role for RIPK1 scaffold functions in the inhibition of necroptosis even when RIPK1 is not an active participant in the necrosome, most likely by sequestering RIPK3 in an inactive state. While the authors did not specifically show that the late-onset skin phenotype was driven by apoptotic death, they did show that primary keratinocytes derived from *Ripk1^EKO^* and from *Ripk1^EKO^ Zbp1^Za1a2/Za1a2^* were sensitised to apoptotic cell death upon treatment with TNF plus cycloheximide [22]. It seems likely that, as in the above mouse models, the late-onset skin phenotype may once again be driven by low-level spontaneous apoptosis precipitated by the loss of RIPK1.

It would be interesting to determine if the necroptotic phenotype of the *Ikk2*^EKO^, *Rela^EKO^cRel^EKO^*, and *Nemo^EKO^* is also driven by nucleic acid-sensing. The question then becomes, what is the triggering event? ZBP1 expression, which is normally restricted to what are likely myeloid cells scattered throughout the dermis, is increased in *Ripk1^EKO^* epidermis [22,131,132]. However, the cornification programme actively prevents extracellular nucleic acids through the complete degradation of DNA by epidermal-specific DNases [30,35]. One hypothesis could be that aberrant epidermal apoptosis is still the initiating factor. As discussed earlier, the active suppression of apoptosis during cornification supports the idea that apoptosis has greater inflammatory potential in the epidermis, perhaps because it is a barrier tissue making it more prone to the retention of apoptotic cells. This allows them to progress to late apoptosis and the release of inflammatory cell contents, including nucleic acids. This could then trigger an inflammatory amplification loop where sensing of nucleic acids can drive the expression of type I and type II interferon-mediated genes, including *Zbp1*. Increased ZBP1 expression and the presence of those same nucleic acids may initiate necroptotic cell death, which drives an even stronger response amplifying the inflammatory effects and producing more extracellular DNA. Unfortunately, because blocking TNFR1-mediated apoptosis will drive RIPK1-mediated necroptosis, simply blocking apoptosis in these models does not prevent disease. It would be interesting, though, to determine if there is a difference in the ratio of various necrosomes depending on whether or not apoptosis is blocked.

### Necroptosis can also limit tissue inflammation

Enhanced inflammation in *Ripk3^−/−^* and *Mlkl^−/−^* at early timepoints in the IAP depletion model shows how necroptotic cell death in the skin may limit tissue inflammation [100]. Similarly, *Mlkl* KOs have increased inflammatory markers, including IL-6, TNF, and IL-1β, upon *Staphylococcus aureus* infection [124]. In this case, necroptosis contributes to improved infection outcomes not by participating in bacterial death but by limiting the damage caused by excessive inflammation. Interestingly, in the same model, *Ripk3^−/−^* had increased bacterial clearance and reduced inflammation. This can be attributed to the involvement of RIPK3 in multiple cell death and inflammatory responses, independent of MLKL. The absence of RIPK3 in this model led to decreased production of IL-1β and activation of apoptosis, which protected the mice from *S. aureus*-induced inflammatory damage [124].

## Pyroptosis

Pyroptosis is thought to have evolved to prevent pathogen replication in cells. Cell death is characterised by pore formation and the release of pro-inflammatory cytokines. Cellular swelling is followed by loss of membrane integrity and the release of cellular contents, including any intracellular pathogens [140–142]. Pyroptosis is typically initiated downstream of inflammasomes. These large multimeric complexes are nucleated by self-oligomerising sensor proteins that form a molecular scaffold to activate inflammatory caspases. Activation and assembly of inflammasome complexes is mediated by DAMPs and PAMPs through specific PRRs [140,142].

**Figure 5.**
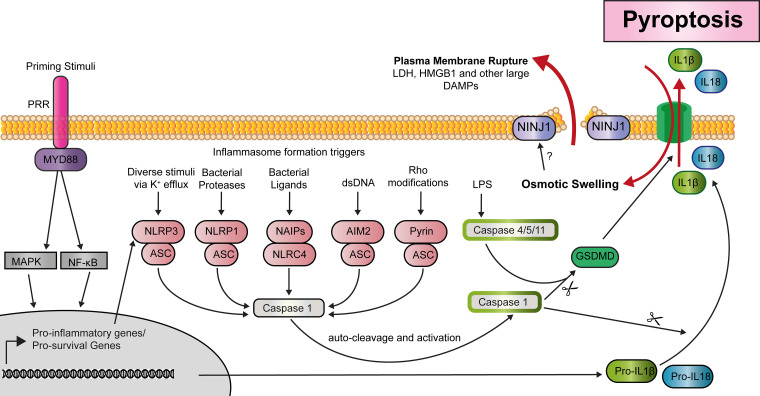
Inflammasome formation and pyroptosis. Pattern recognition receptor (PRRs) signalling via MYD88 can induce activation of MAPKs and NF-κB, which up-regulate the transcription of genes including those encoding pro-IL-1β and NLRP3. A variety of stimuli trigger the formation of the various inflammasome complexes. Inflammasome assembly leads to the autoproteolytic cleavage and activation of caspase-1, which then cleaves a variety of substrates including pro-IL-1β and pro-IL-18, and the pyroptotic effector GSDMD. Cleaved GSDMD binds to lipids in the plasma membrane and forms oligomeric pores, enabling the release of IL-1β and IL-18. Pore formation also results in cellular swelling due to osmotic pressure and to the death of the cell. Plasma membrane rupture will typically follow pore formation however this is not a passive process due to osmotic swelling as long thought but is instead mediated by Ninj1 following osmotic swelling. Membrane rupture results in the release of cell contents too large to fit through the GSDMD pore. Inflammasome-independent pyroptosis occurs when caspase-4 or caspase-5 (caspase-11 in mice) directly binds LPS and triggers self-oligomerisation, activation of the caspases and cleavage of GSDMD. Unlike caspase-1 these caspases do not directly cleave pro-IL1β and pro-IL18.

Inflammasome-independent (non-canonical) pyroptosis occurs when caspase-4 or caspase-5 (caspase-11 in mice) directly binds lipopolysaccharides (LPS), independent of TLR4 (the classic PRR of this ligand) [143]. This binding triggers self-oligomerisation and activation of the caspases without the need for an upstream molecular scaffold [144–146] (Figure 5). Non-canonical pyroptosis occurs upon intracellular invasion by Gram-negative bacteria representing a clear infective trigger that will almost always result in pyroptotic death of the cell.

### Regulation of pattern recognition receptors is an essential component in the skin

Pattern recognition is vital to surveillance and response in the skin. Signalling responses to PRR activation will often involve complex interactions that are in many ways poorly understood. A single PRR can recognise multiple stimuli, and simultaneous signalling within the same cell may be complimentary, amplifying, or inhibitory to the PRR signal. Thus, the downstream response upon activation of these pathways can be highly context dependent. PRRs can activate inflammatory transcription via NF-κB and mitogen-activated protein kinase (MAPK) but also promote inflammasome assembly and the formation of intracellular death complexes [147,148]. Additionally, host monitoring of the microbiota occurs via the TLRs [149–151]. Thus, PRRs operate at the nexus of tolerance, inflammation, and cell death. Proper regulation of these pathways is essential in the skin to ensure a prompt and adequate response upon infection or injury while maintaining tolerance to commensal microorganisms.

### PRRs trigger inflammasome formation

There are several types of inflammasomes defined by their core sensor molecule. The most common are of the Nod-like receptor (NLR) family, though inflammasome complexes can also initiate and form around non-NLR proteins, such as AIM2 or Pyrin [147,152]. The NLRP3 inflammasome is the most well studied. The expression of NLRP3 is highly enriched in macrophages and can be detected at lower levels in other immune cells, including epidermal resident LCs [153–156].

Pyroptosis from this complex occurs via a two-step process. First is a priming step such as engagement of a TLR by a bacterial ligand. This results in the activation of transcription factors, including NF-κB, leading to up-regulation of pro-IL-1β and NLRP3. A second stimulus is later detected by NLRP3, prompting assembly of the inflammasome complex. Caspase-1 is recruited to the complex via the adapter protein ASC (Figure 3) [147,148]. Other inflammasome nucleating molecules, such as NLRP1, AIM2, and NLRC4 (also known as IPAF), do not need a priming step for inflammasome formation. However, priming is typically still required for pro-IL-1β expression prior to its caspase 1-mediated cleavage (Figure 3) [147,157].

### Inflammasome formation can initiate pyroptosis

The NLRs are structurally related to APAF1, the core component of the apoptosome. Similar to APAF1, NLRs promote caspase oligomerisation and activation. Activated caspases cleave various substrates, including nucleases that facilitate DNA fragmentation, one of the hallmark features of pyroptosis. However, unlike in apoptosis, pyroptotic caspases do not limit inflammation [140]. The pyroptotic caspase, caspase-1, cleaves the critical pyroptotic effector molecule, GSDMD [158,159]. Once cleaved, the N-terminal portion of GSDMD binds to lipids in the plasma membrane and forms oligomeric pores. This leads to increased osmotic pressure, cellular swelling, and the death of the cell [158–161] (Figure 3).

### IL-1β and IL-18 secretion

In addition to GSDMD, active caspase-1 also cleaves pro-IL-1β and pro-IL-18, which are then released through pyroptotic pores to the extracellular space [162,163]. There has been some debate as to whether the release of these molecules is directly tied to pyroptotic cell death. Caspase-1 activation is necessary and sufficient for the maturation of IL-1β and is thus a prerequisite for secretion [163]. IL-1β and IL-18 also lack signal peptides for secretion, and thus, pore formation has long been thought to represent their means of release [164]. However, some cells, such as neutrophils, can activate inflammasomes and release these cytokines but are resistant to pyroptotic cell death [165]. One explanation for this is that GSDMD activation and pore formation can induce membrane repair mechanisms in various cell lines. Thus it may be that pyroptosis-resistant cells use the process of inflammasome nucleation and pore formation to secrete IL-1β and IL-18 to drive tissue inflammation while implementing membrane repair mechanisms to prevent death [166].

Casting further doubt on the necessity of pyroptosis for the release of IL-1β, cleaved IL-1β can be secreted in the absence of GSDMD-mediated pore formation, indicating alternative release mechanisms [167–169]. Caspase-1 activation does not automatically trigger pore formation in all cell types. Epithelial cells, including keratinocytes, are resistant to caspase-1-mediated pore formation [140,167,170]. While the efficient, early release of IL-1 β from inflammasome-activated macrophages does appear to be tied to GSDMD pores and pyroptotic cell death [163], slow-release of cleaved IL-1β from macrophages can proceed independently of caspase-1/GSDMD once the mature protein has translocated to the plasma membrane [171].

The resistance of epithelial cells to pore formation and pyroptosis may be protective. Dysregulation of PRRs in barrier tissues could lead to loss of barrier integrity was pyroptosis improperly activated. Additionally, unstimulated human keratinocytes in culture were found to express pro-IL-1β, indicating that keratinocytes do not require priming for its production [172]. PRR signalling in barrier tissues is also responsible for regulating tolerance of the microbiota [173]. As this involves complex interactions between multiple PRR pathways, it makes sense for barrier cells to resist pyroptotic cell death and the associated release of DAMPs, even upon PRR activation and inflammasome formation.

### GSDMD-mediated pyroptosis occurs prior to and independent of plasma membrane rupture

Interestingly, Ninjurin-1 (NINJ1)-mediated rupture of cell membranes follows GSDMD-mediated cell death but has been genetically separated from the upstream events. NINJ1 deficient macrophages still die following inflammasome activation and GSDMD pore formation. The cells exhibit normal GSDMD-mediated IL-1β and IL-18 secretion and morphological changes, including cell swelling. However, the cells fail to rupture, preventing the release of many DAMPs that cannot pass through the GSDMD pore. This includes the prototypical pro-inflammatory DAMP, HMGB1 [98]. These findings are significant in several ways. They show that plasma membrane rupture during pyroptosis is not a passive osmotic process as long presumed. They also uncouple GSDMD-mediated cell death from cell rupture and the wholesale release of DAMPs during pyroptosis.

### The NLRP1 inflammasome modulates UV-induced inflammation but not pyroptosis in human skin

The most highly expressed NLR in human skin is NLRP1, where it appears to be the key inflammasome sensor in the epidermis [174–176]. It is broadly expressed but can be found at particularly high levels in differentiated epithelial cells such as keratinocytes [177]. The NLRP1 inflammasome is triggered by the bacterial ligand muramyl dipeptide (MDP), by the anthrax lethal toxin, and by UV irradiation via intracellular ATP depletion [177]. What is interesting about the latter is that while keratinocytes remain resistant to pyroptosis upon caspase-1 activation, NLRP1 inflammasome formation in human keratinocytes upon UV irradiation can prompt caspase-1 mediated apoptotic cell death instead. However, the inflammasome itself is dispensable for caspase-1-mediated apoptosis to occur [175,178,179]. This supports the idea that barrier tissue resistance to pyroptosis may function to prevent excessive DAMP release into an already highly immunostimulatory environment while still allowing for the appropriate (and largely DAMP-free) death of potentially malignant, replicating keratinocytes upon UV irradiation.

## NETosis

NETosis refers to cell death occurring upon extrusion of neutrophil extracellular traps (NETs), a mesh of decondensed chromatin and histones covered in granular and cytoplasmic proteins [26,180,181]. Despite the name, neutrophils are not the only NET-producing cells. Similar extracellular meshes have been associated with eosinophils, basophils and mast cells [182–185]. The primary function of NETosis appears to be infection control. Antimicrobial agents decorate the NETs, and the mesh will physically confine (trap) pathogens at the site of infection [186–188]. NETs are commonly found in healing wounds where their beneficial effects are linked to their antimicrobial activity [189]. However, NETosis is a highly inflammatory process. The NETs themselves contain autoantigens, and NET extrusion involves cell membrane perforation. NETotic cells with ruptured cell membranes release DAMPs and other inflammatory mediators, thereby exacerbating the inflammatory response and causing tissue pathology [190]. Up-regulation of NETosis in diabetic wounds enhances inflammation and delays healing [191].

### Molecular events associated with the NET formation

The precise mechanisms involved in NET formation have not been fully established. An NADPH oxidative burst and peptidyl arginine deiminase 4 (PAD4)-mediated histone citrullination and chromatin decondensation appear to be necessary steps [192]. The migration of neutrophil elastase (NE) to the nucleus is thought to promote histone processing and chromatin decondensation [193,194]. NE and an alternative neutrophil serine protease (NSP), cathepsin G, can also induce GSDMD cleavage in neutrophils [195,196], which is necessary for NET extrusion [197,198]. Thus, NE has long been considered a key mediator of NETosis. However, the use of specific NSP inhibitors failed to prevent DNA extrusion in neutrophils suggesting that the catalytic activity of NSPs, including NE, is dispensable for NET formation [199].

### Pyroptosis or necroptosis with NETs: is NETosis truly a separate form of PCD?

There is ongoing debate as to whether NETosis constitutes a separate form of cell death [200,201]. Cellular lysis does not occur in all cases of NET extrusion and can involve the selective extrusion of mitochondrial (rather than nuclear) DNA without lytic cell death [185,202]. There is significant evidence of mechanistic overlap between NETosis and pyroptosis. Both processes play a role in infection control and share many similar triggers [140,203,204]. Caspase-11 and GSDMD are required for plasma membrane perforation to enable NET release [197,198], and pyroptosis-related IL-1β has been associated with the triggering of NETosis [205].

NETs have also been observed in neutrophils undergoing necroptosis in both human and mouse cells [206,207]. Treatment of human neutrophils with an MLKL inhibitor (necrosulfonamide (NSA)) reduced NET formation *in vitro* [206]. NET formation and associated activation of RIPK3/MLKL has been observed in neutrophil-rich tissue samples from patients with cutaneous vasculitis and psoriasis [207]. However, necroptotic signalling does not appear to be necessary for NET formation [208].

The key distinction between pyroptosis and NETosis is whether DNA is retained (pyroptosis) or expelled (NETosis) during lysis [199]. However, given the mechanistic overlap, another way of thinking about this could be that NET formation is a parallel process that can (but does not always) occur during the execution of lytic cell death programmes. Whether or not NET formation occurs will depend on the stimuli. Extrusion of the NETs could then be described as a by-product of MLKL or GSDMD pore formation.

## Ferroptosis

Ferroptosis is a biochemically distinct form of regulated cell death. It is triggered when dysregulation of intracellular iron homeostasis leads to an accumulation of iron-dependent reactive oxygen species (ROS). Excess cellular ROS induces lipid peroxidation causing lethal damage to lipids, proteins and nucleic acids, and the caspase and necrosome independent death of the cell [209–211]. The anti-oxidative enzyme Glutathione peroxidase 4 (GPX4) reduces and prevents lipid peroxidation making it a key regulator of ferroptosis. Inhibition or ablation of GPX can be used to induce ferroptosis *in vitro* and *in vivo* [212–214].

Morphological features of ferroptosis include reduced cell volume, shrunken mitochondria, reduction in mitochondria crista, and mitochondrial outer membrane rupture [215], though notably without the release of cytochrome-c or activation of caspases [209]. Ferroptosis can be inhibited by iron chelators, lipophilic antioxidants, and lipid peroxidation inhibitors [216,217].

### Ferroptosis-associated inflammation

Ferroptotic cells are potent mediators of tissue inflammation. The release of DAMPs, including HMGB1, cell-free (cf)DNA and IL33, makes ferroptotic cell death inflammatory. However, the mechanisms of DAMP release are not entirely clear. Cytoplasmic and organelle swelling, and plasma membrane rupture as seen in necroptosis or pyroptosis are not universally observed during ferroptosis [209,218,219]. One reason for this may be the tug of war between lipid peroxidation-induced membrane damage and membrane repair mechanisms. Both processes can be initiated by the same triggers such as ER stress-mediated calcium influx [219,220]. Some degree of membrane damage even without wholesale membrane rupture may allow for the passive release of DAMPs. However, there is evidence to suggest that the active release of certain DAMPs may also occur. One study has shown that, the prototypical DAMP, HMGB1, is actively released during ferroptosis upon its autophagy-dependent acetylation [221].

Additionally, ferroptotic cells can work as signal transmitters inducing a chain of further ferroptosis in surrounding cells in a paracrine effect. Lipid peroxidation and its aftereffects can propagate from ferroptotic cells to surrounding cells that were not exposed to ferroptosis inducers [222].

### The potential role of ferroptosis in the skin

In recent years excessive or defective ferroptosis has been associated with a plethora of disease states, most notably cancer, neurodegeneration, and ischaemic organ injuries [6,223]. Ferroptosis may also contribute to pathogenesis in autoimmune diseases, including SLE [224,225]. Mice with neutrophil-specific Gpx4 haploinsufficiency (*Gpx4*^fl/wt^ LysMCre^+^) develop SLE-like symptoms including autoantibodies, neutropenia, skin lesions, and proteinuria [225]. Until very recently the role of ferroptosis in skin pathophysiology has remained largely unexplored. That said, there are emerging indications of its potential relevance.

Cultured primary keratinocytes treated with the ferroptotic inducer erastin had reduced viability and increased expression of a suite of psoriasis-associated cytokines (*TNF-α*, *IL-6*, *IL-1α*, *IL-1β*, *IL-17*, *IL-22*, and *IL-23*). These effects were reversed when cells were co-treated with Fer-1, a specific inhibitor of ferroptosis. Fer-1 treatment also reduced the severity of imiquimod-induced psoriasis in mice [226].

Ferroptosis appears to contribute significantly to acute inflammation in sunburn. A portion of the UVB radiation-induced death of primary human epithelial keratinocytes (HEKs) in culture could be prevented by treatment with various ferroptotic inhibitors, particularly in the first 6 h following radiation [227]. The death of these cells in the absence of the inhibitors was accompanied by ferropototic death signals, including the accumulation of oxygenated phospholipids, and the release of HMGB1 into the media. HMGB1 release was mitigated by treatment with Fer-1, but not by the apoptotic inhibitor Z-Vad. Mice topically pre-treated with Fer-1 prior to UVB irradiation had reduced immune cell infiltrate and inflammatory cytokines in the skin. This effect was not seen in mice pre-treated with Z-Vad, supporting the idea that a significant portion of UVB-induced inflammation is due to the ferroptotic death of keratinocytes [227].

While ferroptosis is an inflammatory form of cell death, it may in some circumstances have a protective role in the skin. Down-regulation of GPX4, which is considered a hallmark of ferroptosis, appears to contribute to the resolution of skin inflammation and of non-melanoma skin cancer [228]. Additionally, while excessive or prolonged HMGB1-driven inflammation is associated with chronic inflammatory disease [14,229,230], HMGB1 can also stimulate tissue repair processes, including keratinocyte migration and angiogenesis [231–235]. That HMGB1 appears to be actively released during ferroptosis suggests pathophysiological functions for the DAMP in that context. Acute ferroptotic inflammation associated with UVB radiation, for example, may be an important mechanism for promoting epidermal healing in sunburn. Thus, whether its association with any particular skin disease is causative, reactionary, or protective will need to be carefully examined.

## Cell death in human skin disease

Histopathological findings of cell death in patient skin samples are typically described as apoptotic or necrotic, but the precise nature of said necrosis usually remains undefined. This simple dichotomy has been the case for decades. In the meantime, the cell death field has expanded substantially. Late apoptosis, necroptosis, pyroptosis, and NETosis and ferroptosis have all been associated with human skin conditions and could all potentially be described as necrotic by the histopathologist.

### Apoptosis and necroptosis in human skin disease

Epidermal apoptosis features in inflammatory skin conditions in humans, including Lichen planus (LP) [236], atopic dermatitis (AD) [237], graft versus host disease (GvHD) [238], bullous pemphigoid [239], and chronic diabetic skin ulcers [240]. Apoptotic keratinocytes that release DAMPs have been posited as a potentially initiating event in the pyoderma gangrenosum [241]. Activation of the RIPK3–MLKL axis has been observed in some neutrophilic diseases [207] including psoriasis [242]. Increased epidermal expression of RIPK3 has also been seen in the lesional epidermis in lichenoid tissue reactions [243], and in SJS/TEN [244]. Loss of cFLIP in keratinocytes may play a role in the increased caspase-8 mediated cell death seen in TEN/SJS patients [101,245].

### Inflammasomes and pyroptosis in skin-associated disease

Variations in NLRP1 are associated with susceptibility to inflammation and autoimmunity contributing to vitiligo, psoriasis and AD [177,246–248]. Inflammasome activation and IL-1β and IL-18 release are also seen in pyogenic arthritis, pyoderma gangrenosum, and acne (PAPA) syndrome and in hidradenitis suppurativa. In PAPA, mutations in proline–serine–threonine phosphatase-interacting protein 1 (*PSTPIP1*) lead to hyperphosphorylation of PSTPIP1, increasing its interaction with the autoinhibitory domain of the pyrin inflammasome, promoting inflammasome activation and IL-1β production [249]. Activated caspase-1 and increased expression of NLRP3 and IL-18 were seen in skin biopsies from hidradenitis suppurativa patients [250,251], suggesting the potential involvement of pyroptotic cell death in this disease.

### Ferroptosis in inflammatory skin disease

To date, there have been very few studies directly linking ferroptosis with inflammatory skin diseases in humans. However, there is reason to think it may play a role. GPX4 expression was reduced and markers of lipid oxidation were significantly up-regulated in psoriasis patient samples. Lipid oxidation activity in keratinocytes was highly correlated to Th22/Th17 activity, a hallmark of psoriatic inflammation [226]. Elevation of IL-4 and IL-13 are hallmarks of Th2 inflammatory diseases including AD [252]. Increased expression of these molecules suppresses expression of GPX4 [253], which would in turn sensitise cells to ferroptosis. Keratinocyte-derived ROS, increased oxidative stress, and decreased enzymatic and non-enzymatic antioxidants appear to contribute to barrier defect and pathogenesis in AD [254,255]. ROS are also notably elevated in LP [256] and in OLP [257,258] where lipoperoxidation and carbonyl stress are thought to contribute to the progression to carcinogenesis. Thus far these effects have not been identified explicitly as ferroptosis. They do however suggest that further exploration is warranted. The precise role that ferroptosis may play in inflammatory skin disease remains to be seen,

### Multiple inflammatory and cell death programmes contribute to skin disease in complex trait disorders

Disrupted innate immune signalling is a common feature in inflammatory skin disease. As a barrier tissue, the skin is exposed continuously to commensals and pathogens, making it vulnerable to inflammatory consequences. Loss of barrier function in epithelial surfaces can result in prolonged exposure to PAMPs, and a more intense inflammatory milieu. Dysbiosis is a common feature of skin diseases, including psoriasis and AD [149,259–261] and is often thought to contribute to the pathology. The combination of genetic susceptibilities, external triggers, and environmental factors highlights the nature of inflammatory skin diseases as complex trait disorders, where multiple processes may coexist and interact to drive disease.

#### Psoriasis

Psoriasis is a chronic, immune-mediated skin disease. The condition is characterised by clearly defined, dry, red plaques with silvery-white scales, epidermal hyperplasia, and marked parakeratosis [262]. Psoriasis arises due to long-term interactions between keratinocytes and infiltrating, activated immune cells. Defects in the cornification programme, reduced apoptosis, increased PRR signalling and possibly aberrant necroptosis, pyroptosis, ferroptosis and NETosis may all play some role in psoriasis pathogenesis and inflammatory exacerbation.

Twin and family studies have shown an important, though undeniably complex, genetic component to psoriasis. What is striking is that genetic susceptibilities for psoriasis frequently involve genes that encode innate immune components including IL-23 signalling (*IL23R*, *IL12B*, *TRAF3IP2*, *IL23A*, *TYK2*), IFN signalling (*IL28RA*, *IFIH1*, *TYK2*, *RNF114*, SOCS1), NF-κB signalling (*REL*, *TNIP1*, *TRAF3IP2*, *TNFAIP3*, *NFKBIA*, *FBXL19*, *CARD14*, *CARM1*), IL-4/IL-13 signalling (*IL4*, *IL13*), and bacterial or viral responsiveness (*NOS2*, *IL28RA*, *DDX58*, *ELMO1*) [263–266]. The effects of these polymorphisms on interacting immune signalling and cell death pathways is no doubt immensely complex and still largely unexplored.

Infiltration by dendritic cells (DCs) expressing TNFα and iNOS is heavily featured in psoriatic lesions (Harden et al. [265]), and the efficacy of anti-TNF in treating psoriasis highlights its importance in the disease. Psoriatic lesions are notably deficient in apoptosis [267]. However, both NET formation and activation of the RIPK3–MLKL axis have been found in neutrophil-rich psoriatic tissue samples [207,268], and mast cell NETosis (METosis) has been identified as an important source of IL-17 in the disease [183]. Increased circulating and lesional HMGB1 is associated with psoriasis pathogenesis, [269] supporting the potential role of these lytic cell death programmes in exacerbating inflammation in psoriasis.

Defective cornification is a feature of psoriasis. Deleting two members of the late cornified envelope (LCE) gene cluster (*LCE3B* and *LCE3C*) confers significant risk for the development of psoriasis [270]. While initially, these proteins were assumed to contribute to skin barrier function, further investigation showed this was not the case and instead identified them as having a defensin-like antimicrobial activity [271]. IL-37 expression is decreased in psoriasis [272,273], and mutations of *IL36RN* can cause generalised pustular psoriasis [274]. Both of these are among the anti-inflammatory proteins that are up-regulated during cornification [41]. NLRP1, NLRP3, AIM2, caspase-1, IL-1, and IL-18 have also all been identified as elevated in psoriatic skin samples compared with healthy controls.

The increased expression of the DNA sensing protein AIM2 in psoriatic keratinocytes is interesting when considered alongside the down-regulation of DNase1L2 observed in parakeratotic psoriasis lesions [35]. While combined loss of DNase1L2 and DNase2 proteins in mice does lead to DNA retention (parakeratosis) in the stratum corneum, it was insufficient to activate inflammatory pathways [30]. However, in healthy skin, AIM2 expression is restricted to LCs and melanocytes [153]. The combination of parakeratosis and up-regulation of AIM2 in psoriatic keratinocytes may contribute to pathogenic inflammation in disease.

Polymorphisms in *NLRP3* and *CARD8* are associated with increased risk of psoriasis development, while *NLRP1* mutations have been associated with diverse cutaneous inflammatory diseases, including psoriasis, highlighting the critical role of NLRP1 in epidermal biology [246]. All of which point towards a role for epidermal host defence, abnormal and chronic activation of inflammasome complexes, and potentially pyroptotic cell death, in the psoriasis pathology.

#### Eczematous dermatitis

Eczematous disease encompasses several similar disorders, including allergic contact dermatitis (ACD), irritant contact dermatitis (ICD), and AD, which is what is commonly and colloquially known as eczema. These disorders present similar symptoms; a highly pruritic (itchy), often painful, red rash. They are acutely eczematous but can progress to a chronic stage which is dry with thickened skin. [275]. Keratinocyte apoptosis caused by skin-infiltrating T-cells appears to be a key event in the pathogenesis of AD and ACD. Keratinocyte apoptosis was observed in lesional skin affected by AD, ACD, and in patch tests. IFNg-mediated up-regulation of FasR sensitises keratinocytes to T-cell-mediated apoptosis [237].

Defects in cornification cause structural abnormalities of the cornified layer that affect epidermal barrier functions. Loss-of-function mutations in the *Filaggrin* (*FLG*) gene are the most significant genetic risk factor for AD development, though they are neither sufficient nor necessary for disease [276–278]. Polymorphisms in the cornification-associated serine protease inhibitor *SPINK5* have also been associated with dermatitis [279–281].

AIM2 expression can also be detected in keratinocytes in AD and ACD [282]. Genome-wide association studies identified additional disease risk loci, many of which are involved in immune regulation, including *IL4*, *IL4RA*, *IL13*, *RANTES*, *IL18RAP*, *TNFSF6B*, *IL2RA*, *IL7R*, *STAT3*, *NOD1*, *NOD2*, *TLR2*, and *CD14* [283,284]. Immune dysregulation may contribute to an overreactive response to allergens and to the pathogenic skin microbe *S. aureus* [260,261,285].

#### Lichen planus

LP is a common, chronic, inflammatory disease that can affect mucosal and cutaneous sites, including the skin (cutaneous lichen planus, CLP) and oral cavity (oral lichen planus, OLP). The disease is mediated by activated CD8^+^ lymphocyte-induced keratinocyte apoptosis, and the consequent release of pro-inflammatory mediators and chronic lymphocytic infiltrate [236,286]. Increased Fas/FasL expression in basal keratinocytes results in increased epidermal apoptosis [287], while little to no apoptosis in the subepithelial cell infiltrate causes persistence of the inflammatory infiltrate and chronicity of the disease [288].

NF-κB expression is increased in keratinocytes from CLP and OLP compared with healthy tissue; however, more severe manifestations of the disease with erosive lesions have reduced epithelial expression of NF-κB and increased keratinocyte apoptosis, demonstrating how a shift from inflammation to apoptotic cell death can exacerbate disease [236,289]. On the other hand, one theory regarding malignant transformation in OLP is that epithelial cells may develop mechanisms to evade apoptosis and increase their proliferation rate [288]. While this may be an attempt to preserve the epithelial structure in the face of the Fas onslaught, it also, unfortunately, instigates two of the hallmarks of cancer [290].

Expression of the caspase-1 inhibitor, CARD18, which is up-regulated during keratinocyte differentiation and cornification, was strongly reduced in involved skin of CLP patients. CARD18 inhibits caspase-1, preventing pyroptosis and indirectly suppressing the secretion of IL-1β [41,291]. The inflammasome nucleating proteins AIM2 and NLRP1 were also up-regulated in CLP in the epidermis and dermis, respectively [292], again suggesting a potential role for inflammasomes and pyroptosis in enhancing inflammation in LP.

## Therapeutic opportunities for targeting cell death pathways

Immunomodulatory drugs are the cornerstone of treatment for ISDs. In mild to moderate disease, this means topical corticosteroids. If the disease is severe or widespread topical treatments are less practical or effective, indicating the use of systemic immunosuppressant medications such as cyclosporin or methotrexate [293,294]. In the last decade, biologic drugs that target mediators of inflammatory and immune responses have had considerable success in treating a range of inflammatory disorders. However, their action is not universal. The nature of ISDs as complex trait disorders means that the success of any particular treatment can vary widely from person to person. Some patients have no initial response, while others may lose the response over time. A subset of patients also experience a paradoxical induction or exacerbation of cutaneous disease in response to biologic treatment [295–301].

There is the additional consideration that both steroids and non-steroidal immunosuppressant drugs have factors that limit their long-term use [293,302]. These treatments are not a cure, and conditions such as AD and psoriasis can be lifelong. Maintaining patients on systemic immune-suppressing medications such as steroids, anti-TNF, or T-cell modulation therapies means maintaining a lifelong state of immunosuppression.

Given the role that DAMPs can play in enhancing inflammatory feedback loops, there is interest in targeting them to short circuit chronic inflammation. However, there are many potential DAMPs and much uncertainty as to their relative, context dependent, inflammatory potentials. Direct targeting of DAMPs to prevent inflammation, is therefore a difficult proposition. If cell death is a source of DAMPs contributing to chronic inflammation, then inhibiting cell death could be an effective anti-inflammatory approach. Targeting cell death signalling pathways provides an additional therapeutic opportunity for treating inflammatory disease. Small molecule inhibitors targeting critical regulators and inducers of cell death (caspases and RIP kinases) as well as the membrane-disrupting proteins (MLKL, GSDMD and NINJ1) are of considerable interest.

### Targeting RIPKs

A set of small-molecule inhibitors of necroptosis, referred to as necrostatins, were initially identified using unbiased cell-based screening. They were later shown to function by inhibiting RIPK1 kinase functions [303]. As they did not affect the scaffold functions of RIPK1, which, as previously discussed, are required for the formation of molecular platforms during pro-survival signalling, these necrostatins and other RIPK1 targeting drugs could block RIPK1-dependent cell death (both apoptotic and necroptotic) without impairing survival signalling to NF-κB and MAPK. However, early iterations of these drugs had off-target effects, including it seems inhibition of ferroptosis in a RIPK1-independent manner. More selective RIPK1 inhibitors entered human clinical trials for the treatment of IBD, Crohn's disease, RA, ALS, and Alzheimer's disease [303,304]. A randomised control trial of the RIPK1 inhibitor GSK2982772 found use of the drug to be safe and saw some improvements in clinical disease, though the interpretation of results was hampered by an unusually high placebo effect in one of the dosing cohorts. The researchers have suggested that further trials using a higher drug dose and in patients with more active disease are warranted [305,306].

RIPK3 is involved in necroptotic cell death and, in some circumstances, in pyroptosis, apoptosis, and cytokine production. As RIPK3 knockout mice are viable and fertile, RIPK3 inhibition was assumed to be safe and was thus considered a promising treatment target for chronic inflammatory diseases. However, surprisingly, specific inhibition of the RIPK3 kinase domain can be toxic. Mice with a *Ripk3* kinase-dead mutation (*Ripk3^D161N^*) are embryonic lethal due to excessive RIPK1-mediated apoptosis [307]. While several small-molecule inhibitors specific for the RIPK3 kinase domain were developed, they were also found to induce RIPK1-mediated apoptotic cell death [308] and have not progressed through therapeutic development [309]. If interest in RIPK3 inhibition were to be reignited, complete inhibition or protein degradation might be required to recapitulate the inflammatory benefits seen in RIPK3 KO mice.

### Targeting caspases

Caspases are key inducers of extrinsic apoptosis and pyroptosis, both of which are increasingly implicated in inflammatory disease pathogenesis. While this makes them potentially attractive therapeutic targets, there has not been much success with this strategy so far. Targeting specific caspases is difficult due to highly conserved active sites [310], while pan-caspase inhibition risks inhibiting healthy, physiological apoptosis or negating the anti-inflammatory moderation of intrinsic apoptosis. Caspase 8-specific inhibitors might be useful in conditions with excessive extrinsic apoptosis; however, as complete caspase-8 inhibition can trigger necroptosis [311] inhibition would need to be carefully modulated to avoid exacerbating inflammation. Caspase-1 is a promising target for treating IL-1β-mediated conditions. Unlike caspase-8, knockout of caspase-1 in mice is not lethal, so a selective caspase-1 inhibitor is less likely to cause serious off-target effects.

### Targeting membrane permeabilization and cell lysis

MLKL and GSDMD, the membrane-disrupting executioner proteins of necroptosis and pyroptosis, respectively, are also of interest as therapeutic targets [312–314]. As well as their importance in executing those highly inflammatory cell death mechanisms, GSDMD pores are important for the release of IL-1β, and GSDMD and maybe MLKL, are associated with NET extrusion. The MLKL inhibitor NSA blocks MLKL-mediated membrane disruption possibly by affecting oligomerisation, though recent work in epithelial cells showed NSA blocked trafficking of oligomers to the membrane [118]. NSA can also bind to GSDMD, where it appears to affect the formation of the oligomeric pore [312]. NSA, or chemically similar compounds, may be of value in the treatment of IL-1β-mediated conditions, such as PAPA and HS, and in diseases associated with NETosis, including vasculitis, SLE, and psoriasis.

Another potentially attractive target for mitigating inflammation during lytic cell death is NINJ1 [98]. NINJ1 mediates plasma membrane rupture following pyroptotic cell death. It also significantly contributes to membrane rupture in post-apoptotic necrosis and necrosis induced by pore-forming bacterial toxins. A NINJ1 monoclonal antibody prevented plasma membrane rupture in cell culture, indicating that it is effectively targetable [98]. While it would not necessarily be helpful in treating IL-1Β mediated disease as its loss has no impact on GSDMD pore formation and cytokine release, NINJ1 inhibition may have therapeutic benefit in mitigating the wholesale release of DAMPs following pyroptosis or post-apoptotic necrosis.

## Concluding remarks

Inflammation caused by lytic forms of cell death such as necroptosis, pyroptosis and NETosis do have important physiological functions, in particular as part of infection control systems. The physiological importance of ferroptotic inflammation is less clear but it may have a role in immune surveillance and tumour suppression systems [6]. However, dysfunctional innate immune signalling can lead to inappropriate activation of these highly inflammatory processes. The immunostimulatory environment of the skin makes it particularly prone to such over-activation. Even apoptotic cell death, which follows a programme specifically meant to limit or prevent inflammation, can instigate and drive inflammation in the skin. That apoptosis is actively suppressed in keratinocytes during terminal differentiation and cornification indicates the inflammatory potential of apoptotic cells in the epithelial barrier. Accumulating evidence in mouse models and some human studies suggests that targeting cell death has therapeutic potential in inflammatory skin disease. A difficulty remains, however, in the accurate identification of specific cell death modalities in skin disease. Development of good histopathological biomarkers that can distinguish the different cell death modalities is needed if we are to develop better, more targeted treatments.

Inflammatory skin diseases are typically complex trait disorders meaning multiple interacting factors, genetic and environmental, will contribute to disease pathogenesis. While this review has outlined several individual, well-described cell death pathways that can play a role in skin inflammation, the reality is that multiple signalling pathways can coexist and interact in the same disease. Effective treatments may require targeting multiple cell death and inflammatory pathways. That said, a better understanding of the molecular mechanisms that regulate cell death and their specific and interacting contributions to inflammation in the skin has the potential to produce better treatments for inflammatory skin diseases.

## Open Access

Open access for this article was enabled by the participation of University of Melbourne in an all-inclusive *Read & Publish* agreement with Portland Press and the Biochemical Society under a transformative agreement with CAUL.

## Abbreviations

ACD: allergic contact dermatitis
AD: atopic dermatitis
cFLIP: cellular FLICE-like inhibitory protein
CLP: cutaneous lichen planus
DAMPs: damage-associated molecular patterns
EKO: epidermal knockouts
FADD: Fas-associated death domain
GPX4: glutathione peroxidase 4
GSDMD: gasdermin D
HOIL-1: hemeoxidized iron-regulatory protein 2 ubiquitin ligase-1
IAP: inhibitor of apoptosis protein
IKK: IκB kinase
LCs: Langerhans cells
LP: lichen planus
LUBAC: linear ubiquitin-chain assembly complex
MAPK: mitogen-activated protein kinase
MLKL: mixed lineage kinase domain-like
MOMP: mitochondrial outer membrane permeabilisation
NETs: neutrophil extracellular traps
NF-κB: nuclear factor-kappa B
NLR: Nod-like receptor
NSP: neutrophil serine protease
PAMPs: pathogen-associated molecular patterns
PAPA: pyoderma gangrenosum, and acne
PCD: programmed cell death
PRRs: pattern recognition receptors
ROS: reactive oxygen species
SLE: systemic lupus erythema
TAK1: transforming growth factor-β-activated kinase 1
TEN: toxic epidermal necrolysis
TLR: Toll-like receptor
XIAP: X-linked Inhibitor of apoptosis protein
